# Developing a competing risk nomogram that predicts the survival of patients with a primary hepatic neuroendocrine tumor

**DOI:** 10.3389/fmed.2022.960235

**Published:** 2022-11-08

**Authors:** Jianyang Lin, Xiang Li, Xin Ding, Zhihong Chen, Yinyan Wu, Kun Zhao

**Affiliations:** ^1^Department of General Surgery, Affiliated People's Hospital, Jiangsu University, Zhenjiang, China; ^2^Department of Immunology, School of Basic Medicine, Tongji Medical College, Huazhong University of Science and Technology, Wuhan, China; ^3^Department of Neurology, Affiliated People's Hospital, Jiangsu University, Zhenjiang, China

**Keywords:** competing risk model, decision curve analysis, nomogram, primary hepatic neuroendocrine tumor, surveillance

## Abstract

Primary hepatic neuroendocrine tumor (PHNET) is rare liver cancer and related prognostic factors are unclear. The aim of this study was to analyze the prognostic risk factors of patients with PHNETs and establish an assessment model for prognosis. The clinical information of 539 patients with PHNETs who met the criteria for inclusion was extracted from the Surveillance, Epidemiology, and End Results (SEER) database. These patients were randomly assigned to the training (269 cases) and validation sets (270 cases). Prognostic factors in patients with PHNETs were screened using the Cox proportional regression model and Fine–Gray competing risk model. Based on the training set analysis using the Fine–Gray competing risk model, a nomogram was constructed to predict cumulative probabilities for PHNET-specific death. The performance of the nomogram was measured by using receiver operating characteristic curves, the concordance index (C-index), calibration curves, and decision curve analysis (DCA). No differences in clinical baseline characteristics between the training and validation sets were observed, and the Fine–Gray analysis showed that surgery and more than one primary malignancy were associated with a low cumulative probability of PHNET-specific death. The training set nomograms were well-calibrated and had good discriminative ability, and good agreement between predicted and observed survival was observed. Patients with PHNETs with a high-risk score had a significantly increased risk of PHNET-specific death and non-PHNET death. Surgical treatment and the number of primary malignancies were found to be independent protective factors for PHNETs. The competing risk nomogram has high accuracy in predicting disease-specific survival (DSS) for patients with PHNETs, which may help clinicians to develop individualized treatment strategies.

## Introduction

Globally, liver cancer is one of the most common malignancies with the highest morbidity. The most common pathological type is hepatocellular carcinoma, which accounts for ~85% of liver cancer cases (1). A primary hepatic neuroendocrine tumor (PHNET) is a relatively rare pathological subtype that accounts for only 0.11% of primary hepatic tumors and 0.77% of neuroendocrine tumors (NETs) (2, 3). Although the incidence of PHNETs has increased by six times more than that in 1973 (4); they are still rare and predominantly reported in case reports and case reviews (5). At present, their clinical prognosis remains unclear (6).

In the classical Cox regression model, which is commonly used in survival analysis, the end points of the survival outcomes are generally outcome events of interest and censored events (7). However, there are other outcome events that simultaneously occur in the study population, which may prevent or reduce the probability of the outcome events being of interest to the researcher. This kind of event forms a competing relationship with the outcome event of interest and is defined as a competing event. If the competing events were ignored and classical Cox regression was performed directly, the results may be biased. This may result in certain bias and false positives or negatives if the proportion of competing events is >10 or <10%, respectively. The Fine–Gray competing risk model is a more effective model for survival analysis because it allows the survival data end points to be divided into multiple categories and separates competing events from outcome events of interest, thereby eliminating the impact of competing events on prognosis (8, 9).

The current common clinical prognostic evaluation is based mainly on the American Joint Committee on Cancer staging guidelines (10) that include pathological indicators, such as depth of tumor infiltration and the number of lymph node metastases and bloodstream metastases. However, it fails to adequately consider various factors, such as age, race, Primary malignancy number, and other treatments that are considered important for survival, which limits its value and ability to guide an individualized prognosis. A nomogram model, which is based on a large amount of clinical data and uses a combination of independent prognostic factors to quantify individual survival risk, is considered valuable when evaluating patient prognosis (11). In the field of liver cancer, the factors that affect disease-specific survival (DSS) in patients with PHNETs have been analyzed (12), and a nomogram has been constructed to predict the overall survival (OS) (13). However, the use of classical Cox regression for OS analysis may overestimate the cumulative incidence.

This study intended to analyze data from the Surveillance, Epidemiology, and End Results (SEER) database, which is based on a competing risk model, to explore the prognostic factors for PHNETs and construct a nomogram model that provides a basis for individualized patient treatment.

## Methods

### Data collection

The SEER database is an authoritative source for cancer statistics that has detailed the incidence, pathology, treatment, and prognosis of patients in some states and counties within the United States since 1973 (14). The SEER database is characterized by its large volume and relatively comprehensive clinical information. This study used SEER^*^Stat 8.3.9 software (National Cancer Institute, Bethesda, MD, USA) to extract case information of patients with PHNETs. This study did not require informed consent as the SEER dataset is publicly available.

### Inclusion and exclusion criteria

Patients were included in the study if they met the following criteria: the primary tumor was located in the liver at the time of the patient's initial diagnosis; the tumor's pathological histological type was either a large cell neuroendocrine carcinoma, carcinoid tumor, enterochromaffin cell carcinoid, neuroendocrine carcinoma, atypical carcinoid tumor or adenocarcinoma with neuroendocrine differentiation; and the complete clinical, pathological, and follow-up information was available. The patients with a survival time of zero were excluded from the study. Half of the patients were randomly assigned to the training set used to develop the nomogram; the remaining patients served as the validation set.

### Indicators included in the analysis

Covariates in the study included patients' age (<55, 56–66, 67–75, or >76 years), sex, race (white, black, or other), marital status (divorced/separated, married, single/unmarried, or widowed/other), and year of diagnosis (<2003, 2004–2011, or 2012–2016); whether they underwent surgery (yes or no/unknown), radiotherapy (yes or no/unknown), or chemotherapy (yes or no/unknown); the number of regional lymph nodes (0, 1~, or unknown), histological type (carcinoid tumor, neuroendocrine carcinoma, or others), and grade (G1, G2, G3, or Gx); whether it is a primary malignancy (yes/no); the primary malignancy number (1 or 1+); months of survival; outcome (disease-specific death, 1; other death, 2; or survival, 0); and cause of death.

Disease-specific survival was defined as the duration from the date of diagnosis to death from causes of PHNETs. OS was defined as the duration from the date of diagnosis to death or the last follow-up visit, regardless of the cause of death.

### Statistical analyses

R software version 4.1.1 (R Foundation for Statistical Computing, Vienna, Austria) was used for data analysis. Baseline characteristics of both the training and validation sets were compared using the χ^2^ test and Fisher's exact test. The effects of each variable on OS and DSS in patients with PHNET were analyzed with the univariable and multivariable Cox proportional regression models. Survival curves for each variable were plotted using the survival package in the R software based on Kaplan–Meier and log-rank tests, and forest plots were plotted using the forestplot package. PHNET-specific death and non-PHNET death were considered two competing events. The effect of each variable on DSS in patients with PHNETs was analyzed using the cmprsk package of the R language with the Fine–Gray competing risk model (8), where the probability of PHNET-specific death and probability of non-PHNET death were expressed as the cumulative incidence function (CIF). Based on the independent risk factors affecting DSS in patients with PHNETs, we used the regplot package of the R software to construct a nomogram prediction model. The performance of the model was measured using the concordance index (C-index) (15), receiver operating characteristic curve (16), calibration curve (17), and decision curve analysis (DCA) (18). Finally, we divided the training and validation sets into high-risk and low-risk groups (grouped by the median) based on the nomogram and plotted the cumulative risk curves. A *p*-value <0.05 was considered statistically significant.

## Results

### Comparison of clinical baseline characteristics of patients with PHNETs

A total of 539 patients met the inclusion criteria and were randomly divided (5:5) into the training (*N* = 269) and validation sets (*N* = 270). Analysis of the baseline characteristics between the sets showed no statistically significant differences for age, sex, race, marital status, histological type, year of diagnosis, number of regional lymph nodes, history of previous malignancy, grade, radiotherapy, chemotherapy, surgery, primary malignancy number, outcomes (OS, DSS, and PHNET-specific death), and months of survival (Table 1).

**Table 1:** The basic characteristics of PHNET patients in training set and validation set.

**Variables**	**Total (*n* = 539)**	**Train (*n* = 269)**	**Validation (*n* = 270)**	***p***
Age, Median (Q1,Q3)	66 (55, 75)	64 (55, 75)	67 (55, 76)	0.239
**Age.cat**, ***n*** **(%)**				0.247
~55	145 (27)	72 (27)	73 (27)	
56–66	131 (24)	75 (28)	56 (21)	
67–75	131 (24)	61 (23)	70 (26)	
76~	132 (24)	61 (23)	71 (26)	
**Sex**, ***n*** **(%)**				0.093
Female	277 (51)	128 (48)	149 (55)	
Male	262 (49)	141 (52)	121 (45)	
**Race**, ***n*** **(%)**				0.711
Black	76 (14)	36 (13)	40 (15)	
Others	34 (6)	19 (7)	15 (6)	
White	429 (80)	214 (80)	215 (80)	
**Marital**, ***n*** **(%)**				0.121
Divorced/separated	54 (10)	30 (11)	24 (9)	
Married	273 (51)	146 (54)	127 (47)	
Single/unmarried	96 (18)	45 (17)	51 (19)	
Widowed/others	116 (22)	48 (18)	68 (25)	
**Hist.type**, ***n*** **(%)**				0.848
Carcinoid tumor	245 (45)	119 (44)	126 (47)	
Neuroendocrine carcinoma	272 (50)	139 (52)	133 (49)	
Others	22 (4)	11 (4)	11 (4)	
**Diagnosis**, ***n*** **(%)**				0.672
~2003	180 (33)	94 (35)	86 (32)	
2004–2011	230 (43)	110 (41)	120 (44)	
2012–2016	129 (24)	65 (24)	64 (24)	
**Region.nodes**, ***n*** **(%)**				0.392
0	454 (84)	232 (86)	222 (82)	
1~	23 (4)	11 (4)	12 (4)	
Unknown	62 (12)	26 (10)	36 (13)	
**is.primary**, ***n*** **(%)**				0.823
No	135 (25)	69 (26)	66 (24)	
Yes	404 (75)	200 (74)	204 (76)	
**Grade**, ***n*** **(%)**				0.168
G1	91 (17)	39 (14)	52 (19)	
G2	48 (9)	22 (8)	26 (10)	
G3	101 (19)	59 (22)	42 (16)	
Gx	299 (55)	149 (55)	150 (56)	
**Radiotherapy**, ***n*** **(%)**				0.624
No/Unknown	536 (99)	267 (99)	269 (100)	
Yes	3 (1)	2 (1)	1 (0)	
**Chemotherapy**, ***n*** **(%)**				0.144
No/Unknown	387 (72)	185 (69)	202 (75)	
Yes	152 (28)	84 (31)	68 (250)	
**Surgery**, ***n*** **(%)**				0.133
No/Unknown	434 (81)	224 (83)	210 (78)	
Yes	105 (19)	45 (17)	60 (22)	
**Primary number**, ***n*** **(%)**				0.327
1	363 (67)	187 (70)	176 (65)	
1+	176 (33)	82 (30)	94 (35)	
**OS**, ***n*** **(%)**				0.436
0	131 (24)	61 (23)	70 (26)	
1	408 (76)	208 (77)	200 (74)	
**PHNET-specific death**, ***n*** **(%)**				0.61
0	131 (24)	61 (23)	70 (26)	
1	236 (44)	118 (44)	118 (44)	
2	172 (32)	90 (33)	82 (30)	
**DSS**, ***n*** **(%)**				1
0	303 (56)	151 (56)	152 (56)	
1	236 (44)	118 (44)	118 (44)	
Survival.months, median (Q1,Q3)	19 (5, 55)	19 (5, 52)	21 (5, 61.5)	0.451

### Univariable and multivariable Cox analyses for PHNET prognosis

**Figure 1:**
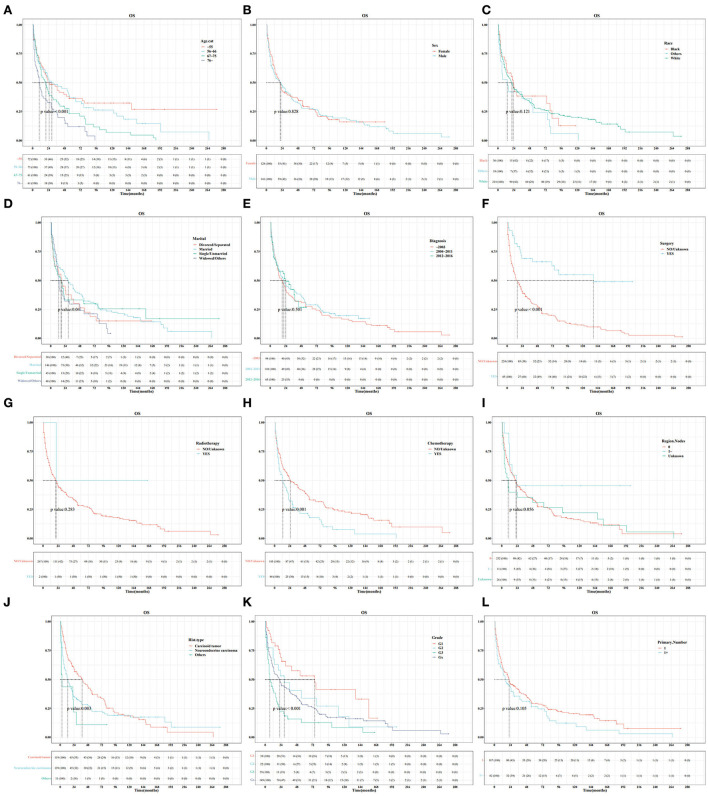
Survival curve analysis of the influence of the main variables on the overall survival (OS) of patients with primary hepatic neuroendocrine tumor (PHNET) in the training set. **(A)** age; **(B)** sex; **(C)** race; **(D)** marital status; **(E)** year of diagnosis; **(F)** surgery; **(G)** radiotherapy; **(H)** chemotherapy; **(I)** number of regional lymph nodes; **(J)** histological type; **(K)** grade; and **(L)** number of primary malignancies.

**Figure 2:**
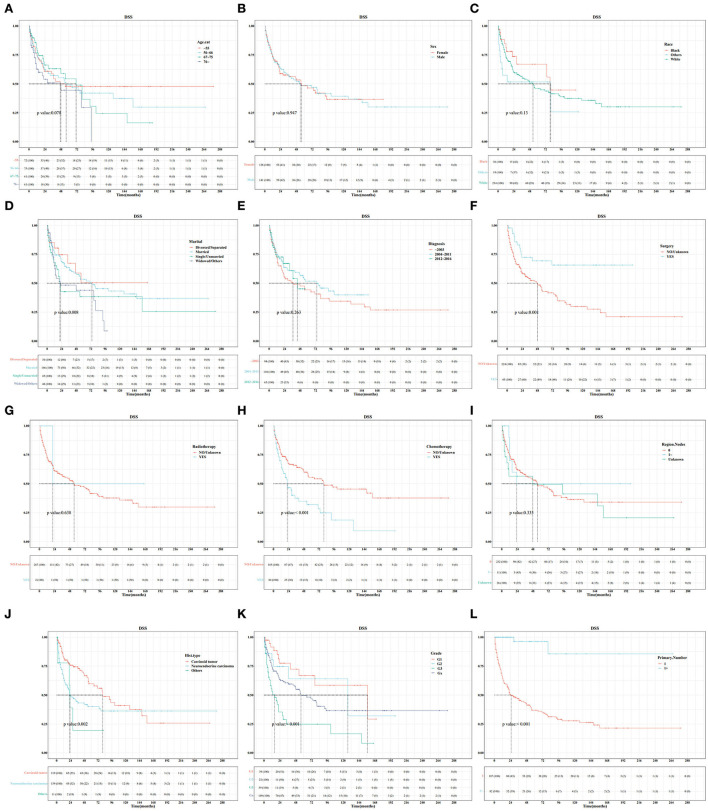
Survival curve analysis of the influence of the main variables on the DSS of patients with primary hepatic neuroendocrine tumor (PHNET) in the training set. **(A)** age; **(B)** sex; **(C)** race; **(D)** marital status; **(E)** year of diagnosis; **(F)** surgery; **(G)** radiotherapy; **(H)** chemotherapy; **(I)** number of regional lymph nodes; **(J)** histological type; **(K)** grade; and **(L)** number of primary malignancies.

In the training set, Kaplan–Meier and log-rank tests were used to plot OS curves for each indicator (Figure 1). Further, analysis with univariable and multivariable Cox proportional regression models showed that older age (67–75 and >76 years), neuroendocrine carcinoma/others, and chemotherapy (yes) were independent risk factors for poorer OS. Conversely, married, widowed/other, and surgery (yes) were independent protective factors for better OS (Table 2). In terms of indicators affecting the DSS of patients with PHNET, Kaplan–Meier and log-rank tests were used to plot DSS curves for each indicator (Figure 2) and found older age (>76 years), other races, neuroendocrine carcinoma, and chemotherapy (yes) to be independent risk factors; whereas, surgery (yes) and higher primary malignancy number were independent protective factors (Table 3).

**Table 2:** Univariate and multivariate Cox analysis of factors for OS in patients with PHNET in the training set.

**Risk factors**	***N* (%)**	**Univariate analysis**		**Multivariate analysis**	
		**HR (95% CI)**	***P*-value**	**HR (95% CI)**	***P*-value**
Age.cat: 56~66 vs. ~55	75 (27.9)	1.178 (0.799–1.738)	0.408	1.084 (0.707–1.661)	0.712
Age.cat: 67~75 vs. ~55	61 (22.7)	1.694 (1.14–2.517)	0.009	1.85 (1.149–2.98)	0.011
Age.cat: 76~ vs. ~55	61 (22.7)	2.349 (1.563–3.531)	<0.001	2.476 (1.492–4.11)	<0.001
Sex: Male vs. Female	141 (52.4)	1.031 (0.782–1.359)	0.829	0.983 (0.722–1.339)	0.913
Race: Others vs. Black	19 (7.1)	1.479 (0.806–2.713)	0.206	1.706 (0.87–3.346)	0.12
Race: White vs. Black	214 (79.6)	1.011 (0.662–1.543)	0.96	1.298 (0.821–2.051)	0.264
Marital: Married vs. Divorced/separated	146 (54.3)	0.781 (0.501–1.215)	0.272	0.484 (0.293–0.799)	0.005
Marital: Single/unmarried vs. Divorced/separated	45 (16.7)	0.874 (0.514–1.487)	0.62	0.785 (0.446–1.382)	0.401
Marital: Widowed/others vs. Divorced/separated	48 (17.8)	1.115 (0.672–1.852)	0.673	0.514 (0.283–0.933)	0.029
Hist.type: Neuroendocrine carcinoma vs. Carcinoid tumor	139 (51.7)	1.378 (1.041–1.823)	0.025	1.566 (1.118–2.195)	0.009
Hist.type: Others vs. Carcinoid tumor	11 (4.1)	2.371 (1.191–4.717)	0.014	2.646 (1.169–5.988)	0.02
Diagnosis: 2004–2011 vs. ~2003	110 (40.9)	0.902 (0.666–1.224)	0.509	1.055 (0.752–1.478)	0.758
Diagnosis: 2012–2016 vs. ~2003	65 (24.2)	0.921 (0.614–1.382)	0.692	0.902 (0.572–1.422)	0.656
Region.Nodes: 1~ vs. 0	11 (4.1)	0.477 (0.211–1.082)	0.077	2.137 (0.774–5.898)	0.143
Region.Nodes: Unknown vs. 0	26 (9.7)	1.109 (0.711–1.729)	0.65	1.067 (0.654–1.74)	0.796
Grade: G2 vs. G1	22 (8.2)	1.64 (0.843–3.194)	0.145	1.082 (0.538–2.175)	0.826
Grade: G3 vs. G1	59 (21.9)	3.313 (1.944–5.645)	<0.001	1.616 (0.898–2.909)	0.109
Grade: Gx vs. G1	149 (55.4)	1.912 (1.179–3.102)	0.009	1.212 (0.724–2.027)	0.464
Radiotherapy: yes vs. No/unknown	2 (0.7)	0.357 (0.05–2.551)	0.304	0.572 (0.069–4.753)	0.605
Chemotherapy: yes vs. No/unknown	84 (31.2)	1.617 (1.208–2.165)	0.001	1.563 (1.125–2.173)	0.008
Surgery: yes vs. No/unknown	45 (16.7)	0.29 (0.178–0.471)	<0.001	0.258 (0.137–0.486)	<0.001
Primary.Number: 1+ vs. 1	82 (30.5)	1.275 (0.953–1.705)	0.102	1.106 (0.809–1.513)	0.527

**Table 3:** Univariate and multivariate Cox analysis of factors for DSS in patients with PHNET in the validation set.

**Risk factors**		**Univariate analysis**		**Multivariate analysis**	
	***n* (%)**	**HR (95% CI)**	***P*-value**	**HR (95% CI)**	***P*-value**
Age.cat: 56~66 vs. ~55	75 (27.9)	1.126 (0.699–1.815)	0.626	1.223 (0.729–2.05)	0.446
Age.cat: 67~75 vs. ~55	61 (22.7)	1.113 (0.656–1.887)	0.692	1.632 (0.881–3.025)	0.12
Age.cat: 76~ vs. ~55	61 (22.7)	1.583 (0.927–2.703)	0.092	2.512 (1.297–4.863)	0.006
Sex: Male vs. Female	141 (52.4)	0.987 (0.687–1.419)	0.944	0.923 (0.602–1.413)	0.711
Race: Others vs. Black	19 (7.1)	1.933 (0.82–4.555)	0.132	3.383 (1.255–9.118)	0.016
Race: White vs. Black	214 (79.6)	1.418 (0.759–2.651)	0.274	1.634 (0.826–3.231)	0.158
Marital: Married vs. Divorced/separated	146 (54.3)	1.161 (0.576–2.34)	0.677	0.698 (0.319–1.525)	0.367
Marital: Single/unmarried vs. Divorced/separated	45 (16.7)	1.763 (0.815–3.812)	0.15	1.091 (0.481–2.477)	0.835
Marital: Widowed/others vs. Divorced/separated	48 (17.8)	1.928 (0.902–4.119)	0.09	0.713 (0.285–1.784)	0.469
Hist.type: Neuroendocrine carcinoma vs. Carcinoid tumor	139 (51.7)	1.707 (1.171–2.487)	0.005	1.734 (1.097–2.74)	0.019
Hist.type: Others vs. Carcinoid tumor	11 (4.1)	2.457 (0.973–6.207)	0.057	1.729 (0.587–5.093)	0.32
Diagnosis: 2004–2011 vs. ~2003	110 (40.9)	0.802 (0.538–1.197)	0.281	0.912 (0.584–1.423)	0.684
Diagnosis: 2012–2016 vs. ~2003	65 (24.2)	0.832 (0.49–1.413)	0.496	0.911 (0.5–1.66)	0.762
Region.Nodes: 1~ vs. 0	11 (4.1)	0.759 (0.308–1.87)	0.549	2.238 (0.658–7.607)	0.197
Region.Nodes: Unknown vs. 0	26 (9.7)	1.233 (0.688–2.21)	0.481	0.941 (0.498–1.777)	0.851
Grade: G2 vs. G1	22 (8.2)	1.273 (0.494–3.285)	0.617	0.779 (0.274–2.211)	0.638
Grade: G3 vs. G1	59 (21.9)	3.993 (2.015–7.913)	<0.001	1.993 (0.914–4.342)	0.083
Grade: Gx vs. G1	149 (55.4)	1.821 (0.962–3.449)	0.066	1.284 (0.645–2.555)	0.477
Radiotherapy: yes vs. No/unknown	2 (0.7)	0.625 (0.087–4.498)	0.641	0.6 (0.064–5.61)	0.654
Chemotherapy: yes vs. No/unknown	84 (31.2)	1.957 (1.343–2.85)	<0.001	1.682 (1.089–2.598)	0.019
Surgery: yes vs. No/unknown	45 (16.7)	0.383 (0.214–0.684)	0.001	0.293 (0.128–0.671)	0.004
Primary.Number: 1+ vs. 1	82 (30.5)	0.045 (0.011–0.181)	<0.001	0.037 (0.009–0.151)	<0.001

### Univariable and multivariable Fine–Gray analyses for PHNET prognosis

**Figure 3:**
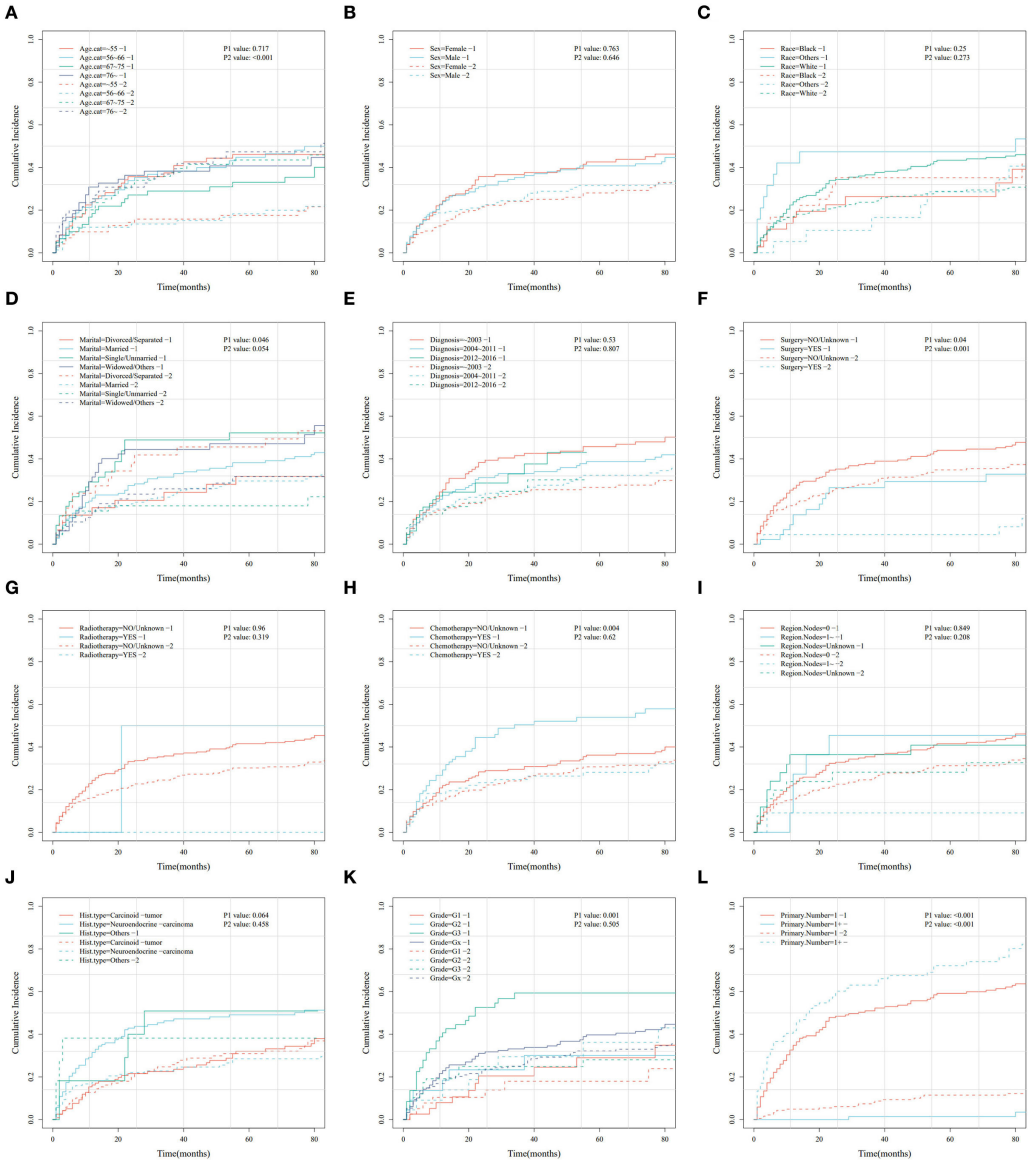
Cumulative risk curve analysis of the influence of the main variables on the cumulative probabilities for primary hepatic neuroendocrine tumor (PHNET)-specific death (labeled by 1) and non-PHNET death (labeled by 2) in the training set. **(A)** age; **(B)** sex; **(C)** race; **(D)** marital status; **(E)** year of diagnosis; **(F)** surgery; **(G)** radiotherapy; **(H)** chemotherapy; **(I)** number of regional lymph nodes; **(J)** histological type; **(K)** grade; and **(L)** number of primary malignancies.

In this study, the cumulative probabilities for PHNET-specific death and non-PHNET death were also analyzed using the Fine–Gray competing risk model. In the training set, CIF curves were plotted for each indicator (Figure 3) and revealed that patients with PHNETs with older age had significantly increased risks of non-PHNET-related death. Further, patients with PHNETs with an advanced grade had a significantly increased risk of PHNET-specific death, divorced patients with PHNETs had a significantly decreased risk of PHNET-specific death, and patients with PHNETs with more primary malignancies had a significantly decreased risk of PHNET-specific death and an increased risk of non-PHNET death. In addition, patients with PHNET without surgical treatment had a significantly increased risk of both PHNET-specific death and non-PHNET death, and patients with PHNET who received chemotherapy had a significantly increased risk of PHNET-specific death.

Moreover, the univariate and multivariate analyses showed that two factors in the training set, older age (>76 years) and other races, were independent risk factors; however, surgery (yes) and the number of primary malignancies were independent protective factors (Table 4). Further, in the validation set, male sex, neuroendocrine carcinomas, grade G3, and radiotherapy treatment were associated with higher cumulative probabilities of PHNET-specific death; in contrast, surgery and more than one primary malignancy were associated with lower cumulative probabilities of PHNET-specific death (Table 5).

**Table 4:** Univariate and multivariate Fine-Gray analysis of factors for cumulative probabilities of PHNET-specific death in the training set.

**Risk factors**	**Univariate analysis**		**Multivariate analysis**	
	**SHR (95% CI)**	***P*-value**	**SHR (95% CI)**	***P*-value**
Age.cat: 56~66 vs. ~55	1.119 (0.699–1.792)	0.64	1.14 (0.691–1.881)	0.61
Age.cat: 67~75 vs. ~55	0.872 (0.522–1.457)	0.6	1.128 (0.578–2.202)	0.72
Age.cat: 76~ vs. ~55	1.017 (0.594–1.742)	0.95	1.917 (0.968–3.799)	0.062
Sex: Male vs. Female	0.969 (0.679–1.382)	0.86	0.906 (0.598–1.373)	0.64
Race: Others vs. Black	2.126 (0.85–5.321)	0.11	2.745 (1.055–7.141)	0.038
Race: White vs. Black	1.557 (0.843–2.875)	0.16	1.469 (0.832–2.592)	0.18
Marital: Married vs. Divorced/separated	1.45 (0.716–2.939)	0.3	1.078 (0.488–2.386)	0.85
Marital: Single/unmarried vs. Divorced/separated	2.142 (0.98–4.685)	0.056	1.428 (0.621–3.285)	0.4
Marital: Widowed/others vs. Divorced/separated	2.145 (1.005–4.58)	0.049	1.195 (0.453–3.153)	0.72
Hist.type: Neuroendocrine carcinoma vs. Carcinoid tumor	1.524 (1.059–2.194)	0.023	1.707 (1.088–2.679)	0.02
Hist.type: Others vs. Carcinoid tumor	1.449 (0.589–3.568)	0.42	2.01 (0.726–5.562)	0.18
Diagnosis: 2004–2011 vs. ~2003	0.771 (0.524–1.134)	0.19	0.818 (0.516–1.298)	0.39
Diagnosis: 2012–2016 vs. ~2003	0.704 (0.42–1.179)	0.18	0.863 (0.474–1.572)	0.63
Region.nodes: 1~ vs. 0	0.982 (0.414–2.331)	0.97	1.913 (0.642–5.703)	0.24
Region.nodes: Unknown vs. 0	1.224 (0.691–2.169)	0.49	1.049 (0.536–2.054)	0.89
Grade: G2 vs. G1	1.127 (0.446–2.85)	0.8	0.852 (0.325–2.233)	0.74
Grade: G3 vs. G1	2.797 (1.467–5.334)	0.002	1.911 (0.94–3.884)	0.073
Grade: Gx vs. G1	1.56 (0.856–2.843)	0.15	1.313 (0.723–2.386)	0.37
Radiotherapy: yes vs. No/unknown	0.954 (0.151–6.011)	0.96	0.835 (0.09–7.786)	0.87
Chemotherapy: yes vs. No/unknown	1.673 (1.164–2.406)	0.005	1.536 (1.001–2.358)	0.05
Surgery: yes vs. No/unknown	0.555 (0.317–0.973)	0.04	0.36 (0.156–0.832)	0.017
Primary number: 1+ vs. 1	0.025 (0.006–0.099)	<0.001	0.018 (0.004–0.074)	<0.001

**Table 5:** Univariate and multivariate Fine-Gray analysis of factors for cumulative probabilities of PHNET-specific death in the validation set.

**Risk factors**	**Univariate analysis**		**Multivariate analysis**	
	**SHR (95% CI)**	***P*-value**	**SHR (95% CI)**	***P*-value**
Age.cat: 56~66 vs. ~55	0.977 (0.598–1.594)	0.92	0.959 (0.579–1.589)	0.87
Age.cat: 67~75 vs. ~55	1.155 (0.72–1.853)	0.55	1.39 (0.844–2.291)	0.2
Age.cat: 76~ vs. ~55	0.99 (0.604–1.621)	0.97	1.359 (0.748–2.472)	0.31
Sex: Male vs. Female	1.137 (0.796–1.624)	0.48	1.466 (1.014–2.12)	0.042
Race: Others vs. Black	2.796 (1.141–6.85)	0.025	1.169 (0.42–3.254)	0.77
Race: White vs. Black	1.138 (0.691–1.874)	0.61	1.086 (0.608–1.941)	0.78
Marital: Married vs. Divorced/separated	1.188 (0.561–2.513)	0.65	1.075 (0.515–2.247)	0.85
Marital: Single/unmarried vs. Divorced/separated	1.698 (0.765–3.767)	0.19	1.277 (0.576–2.83)	0.55
Marital: Widowed/others vs. Divorced/separated	1.704 (0.781–3.72)	0.18	1.887 (0.887–4.015)	0.099
Hist.type: Neuroendocrine carcinoma vs. Carcinoid tumor	1.816 (1.267–2.605)	0.001	1.771 (1.209–2.594)	0.003
Hist.type: Others vs. Carcinoid tumor	0.9 (0.275–2.945)	0.86	0.618 (0.115–3.312)	0.57
Diagnosis: 2004–2011 vs. ~2003	0.848 (0.586–1.226)	0.38	0.801 (0.532–1.204)	0.29
Diagnosis: 2012–2016 vs. ~2003	0.53 (0.293–0.96)	0.036	0.908 (0.51–1.615)	0.74
Region.nodes: 1~ vs. 0	0.446 (0.154–1.291)	0.14	1.601 (0.47–5.457)	0.45
Region.nodes: Unknown vs. 0	0.816 (0.494–1.348)	0.43	0.614 (0.372–1.014)	0.057
Grade: G2 vs. G1	0.814 (0.344–1.928)	0.64	1.041 (0.457–2.374)	0.92
Grade: G3 vs. G1	3.416 (1.932–6.038)	<0.001	3.008 (1.561–5.793)	<0.001
Grade: Gx vs. G1	1.25 (0.769–2.03)	0.37	1.114 (0.664–1.867)	0.68
Radiotherapy: yes vs. No/unknown	2.411 (1.953–2.976)	<0.001	8.048 (1.027–63.076)	0.047
Chemotherapy: yes vs. No/unknown	1.991 (1.363–2.908)	<0.001	1.413 (0.939–2.126)	0.098
Surgery: yes vs. No/unknown	0.403 (0.244–0.666)	<0.001	0.298 (0.154–0.575)	<0.001
primary.number: 1+ vs. 1	0.103 (0.053–0.2)	<0.001	0.077 (0.035–0.169)	<0.001

### Construction and evaluation of a competing risk nomogram model for PHNETs

**Figure 4:**
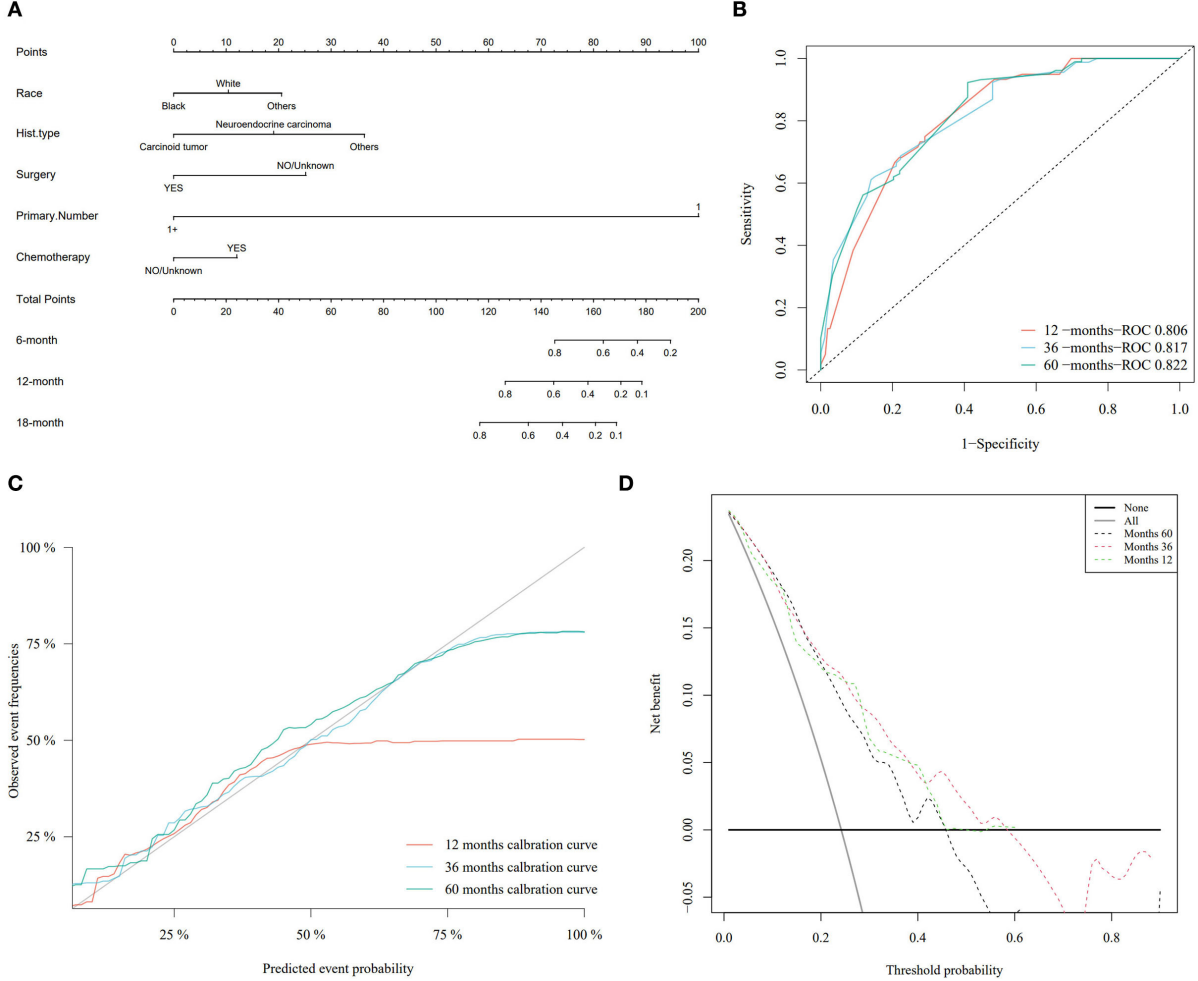
Nomogram model and its performance based on Fine–Gray analysis of patients with primary hepatic neuroendocrine tumor (PHNET) in the training set. **(A)** Nomogram model; **(B)** receiver operating characteristic curve; **(C)** calibration curves; and **(D)** decision curve analysis.

**Figure 5:**
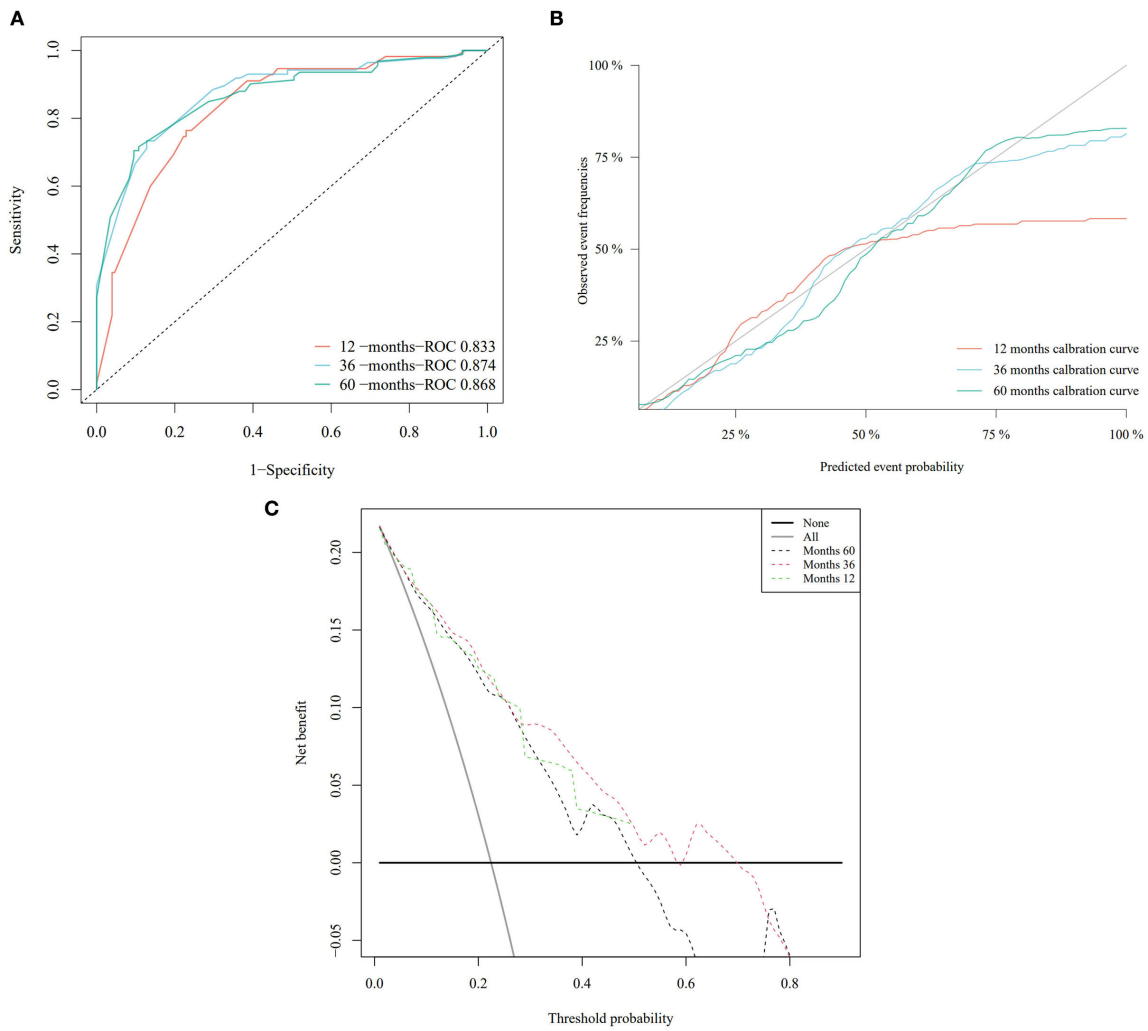
The performance of the nomogram model was evaluated with the validation set. **(A)** Receiver operating characteristic curve; **(B)** calibration curves; and **(C)** decision curve analysis.

**Figure 6:**
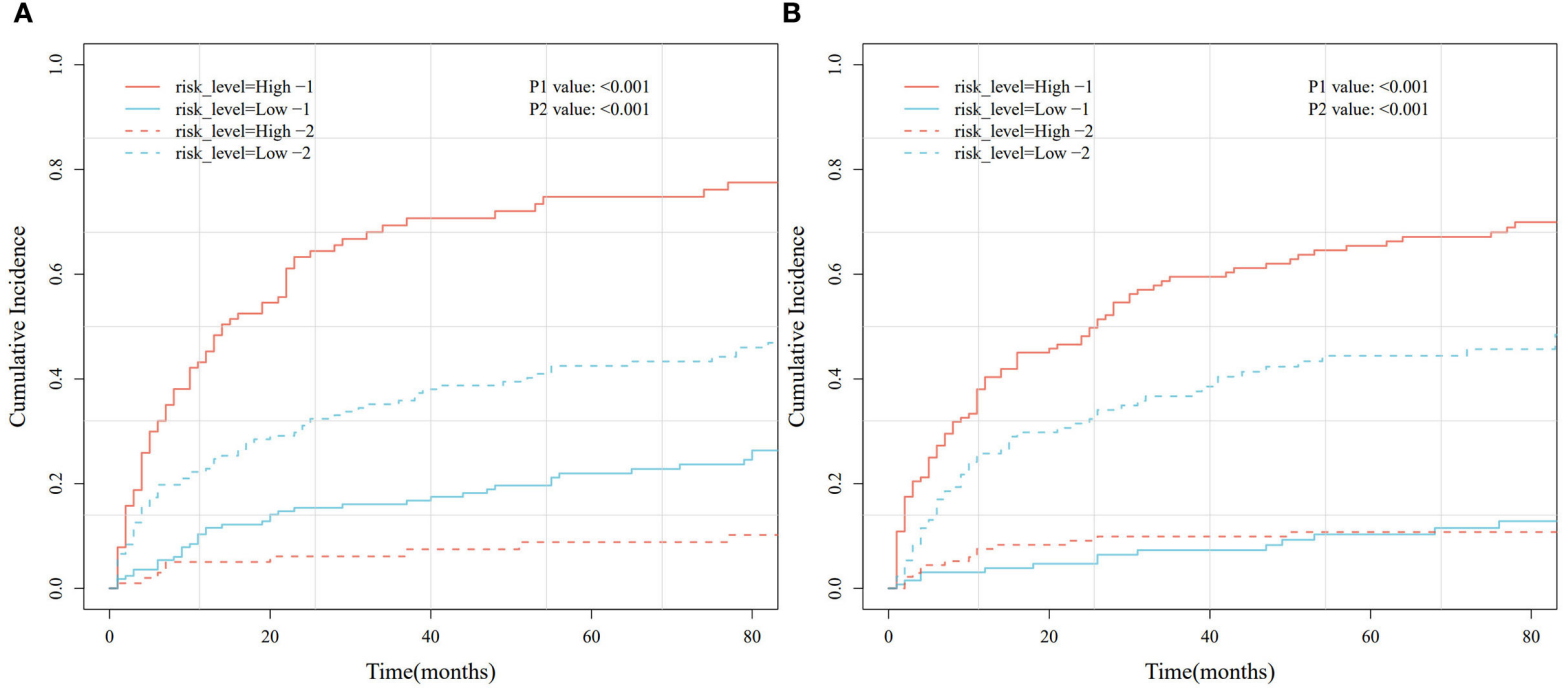
Cumulative risk curve analysis based on different risk groups generated by the nomogram model in the training and validation sets. **(A)** Training set. **(B)** Validation set.

Based on the Fine–Gray analysis of the training set, five statistically significant indicators (multivariate analysis) were included in a competing risk nomogram model to predict the 1-, 3-, and 5-year cumulative probabilities for PHNET-specific death (Figure 4A). The nomogram demonstrated good discrimination with a high C-index in the training (0.792, 0.799, and 0.799, respectively) and validation sets (0.809, 0.813, and 0.809, respectively) for the 1-, 3-, and 5-year cumulative probabilities. Furthermore, the area under the curve (AUC) values of the nomogram model for predicting the 1-, 3-, and 5-year cumulative probabilities for PHNET-specific death were 0.799, 0.818, and 0.829, respectively, in the training set and 0.833, 0.875, and 0.87, respectively, in the validation set (Figures 4B, 5A). The calibration curves for the 3- and 5-year cumulative probabilities were similar to the standard curves in both the training and validation sets (Figures 4C, 5B). Furthermore, all decision curve analyses showed that the nomogram illustrated a high net benefit (Figures 4D, 5C). Further, based on the total score of each patient generated by the nomogram in the training set, all patients were divided into either a low- or high-risk group. The Fine–Gray analyses depicted that the high-risk group had higher and lower cumulative probabilities of PHNET-specific death and non-PHNET death, respectively, than the low-risk group (Figure 6).

## Discussion

Neuroendocrine tumors are a heterogeneous group of tumors originating from peptidergic neurons and neuroendocrine cells that can occur throughout the body, although incidences in the liver are rare (19, 20). In this study, independent risk factors affecting the DSS of 539 patients with PHNET were identified by a multivariable Cox regression model and Fine–Gray competing risk model. Based on race, surgical and chemotherapy treatments, histological tumor type, and the number of primary malignancies, a nomogram was drawn to predict the cumulative probability of PHNET-specific death.

The multivariate Cox analysis in this study showed that older age (>76 years), neuroendocrine carcinoma, and chemotherapy treatment predicted poorer OS and DSS, while surgery predicted better OS and DSS. It is known that a PHNET is generally diagnosed at an older age (21); however, a case of a PHNET that occurred at a young age, which was misdiagnosed as hepatocellular carcinoma preoperatively, was reported in South Korea (22). This report was consistent with our results that age is an independent risk factor affecting the prognosis of patients with PHNETs. Interestingly, chemotherapy treatment was an independent risk factor that conflicted with a previous case report stating that postoperative platinum-based chemotherapy was recommended in the management of PHNETs (23). This contradiction may be derived from Cox analysis inherent bias. Considering this bias, we performed Fine–Gray analysis, which identified surgical treatment and the number of primary malignancies as independent protective factors in both the training and validation sets. According to Jung et al., surgical treatment is recommended because of the favorable postresection outcomes (24). However, the result that the number of primary malignancies was a protective factor is puzzling, and perhaps more samples should be included for clarification.

A clinical nomogram is a valid and reliable prediction model that combines multiple prognostic factors to accurately calculate and predict individual survival (25). Nomograms have been used extensively in many previous studies, such as those on oropharyngeal cancer (26), young breast cancer (27), low-grade endometrial stromal sarcoma (28), and both malignant pheochromocytoma and paraganglioma (29). In terms of liver cancer, a nomogram has been constructed to predict OS in PHNET cases by Zhang et al. (13); however, this report did not take into account the effect of competing events on PHNET-specific death, which lowered the confidence of the results. In this study, we utilized the Fine–Gray competing risk model to analyze the independent prognostic factors and to construct a nomogram model. Multiple evaluation indicators implied that the nomogram for PHNETs performed well. To further validate the performance of the nomogram model constructed for the training set, we measured the C-index and AUC value and plotted both calibration curves and a decision curve. The results of the validation set were similar to those of the training set, which suggested that our nomogram model effectively predicted the DSS of patients with PHNETs.

This study has some limitations, mostly related to the data from the SEER database. First, most of the data from the SEER database are clinical indicators that do not include laboratory tests, imaging tests, or other indicators; as a result, the nomogram constructed in this study could only incorporate the clinical indicators of patients with PHNETs. Moreover, some clinical indicators in the SEER database were missing, which may have affected the accuracy of the prognostic model constructed in this study. Therefore, more clinical studies are needed to further validate the model.

In conclusion, this study developed a nomogram model to predict the DSS in patients with PHNETs using the clinical data from the SEER database and Fine–Gray analysis. We found that surgical treatment and the number of primary malignancies were independent protective factors for PHNETs, which can be used as an important reference for future decisions regarding clinical treatment.

## Novelty and impact

This study provides a novel, well-calibrated, and good discriminatory competing risk nomogram that has a high accuracy to predict disease-specific survival (DSS) for patients with primary hepatic neuroendocrine tumor (PHNET) that may help clinicians to develop individualized treatment strategies. Surgical treatment and the number of primary malignancies were independent protective factors for PHNET.

## Data availability statement

The original contributions presented in the study are included in the article/supplementary material, further inquiries can be directed to the corresponding author.

## Author contributions

JL, XL, and KZ: study design. YW and XD: collection of patients' clinicopathological data. JL and XL: data assembly, acquisition of survival predictions, and data analysis. KZ and ZC: study preparation and approval. All authors have read and approved the final manuscript.

## Funding

This work was supported by the research foundation of Affiliated People's Hospital of Jiangsu University; the support was granted to KZ and JL (Y2020003-S and Y2019014).

## Conflict of interest

The authors declare that the research was conducted in the absence of any commercial or financial relationships that could be construed as a potential conflict of interest.

## Publisher's note

All claims expressed in this article are solely those of the authors and do not necessarily represent those of their affiliated organizations, or those of the publisher, the editors and the reviewers. Any product that may be evaluated in this article, or claim that may be made by its manufacturer, is not guaranteed or endorsed by the publisher.

## Abbreviations

AUC: the area under the curve
CIF: cumulative incidence function
C-index: concordance index
DCA: decision curve analysis
DSS: disease-specific survival
NET: neuroendocrine tumors
OS: overall survival
PHNET: primary hepatic neuroendocrine tumor
ROC: receiver operating characteristic
SEER: Surveillance, Epidemiology, and End Results.
